# Head and neck squamous cancer progression is marked by CLIC4 attenuation in tumor epithelium and reciprocal stromal upregulation of miR-142-3p, a novel post-transcriptional regulator of *CLIC4*

**DOI:** 10.18632/oncotarget.27387

**Published:** 2019-12-31

**Authors:** Brandi L. Carofino, Kayla M. Dinshaw, Pui Yan Ho, Christophe Cataisson, Aleksandra M. Michalowski, Andrew Ryscavage, Addie Alkhas, Nathan W. Wong, Vishal Koparde, Stuart H. Yuspa

**Affiliations:** ^1^Laboratory of Cancer Biology and Genetics, Center for Cancer Research, National Cancer Institute, Bethesda, MD, USA; ^2^Department of Molecular and Cellular Biology, University of California, Berkeley, Berkeley, CA, USA; ^3^Department of Pediatrics, Division of Stem Cell Transplantation and Regenerative Medicine, Stanford University School of Medicine, Stanford, CA, USA; ^4^Oncology-Gynecology, Park Ridge, IL, USA; ^5^CCR Collaborative Bioinformatics Resource (CCBR), Center for Cancer Research, National Cancer Institute, Bethesda, MD, USA; ^6^Advanced Biomedical Computational Science, Frederick National Laboratory for Cancer Research, Frederick, MD, USA

**Keywords:** in situ hybridization, single-cell RNA-sequencing, stroma, miRNA, immune infiltration

## Abstract

Chloride intracellular channel 4 (CLIC4) is a tumor suppressor implicated in processes including growth arrest, differentiation, and apoptosis. CLIC4 protein expression is diminished in the tumor parenchyma during progression in squamous cell carcinoma (SCC) and other neoplasms, but the underlying mechanisms have not been identified. Data from The Cancer Genome Atlas suggest this is not driven by genomic alterations. However, screening and functional assays identified miR-142-3p as a regulator of *CLIC4*. *CLIC4* and miR-142-3p expression are inversely correlated in head and neck (HN) SCC and cervical SCC, particularly in advanced stage cancers. *In situ* localization revealed that stromal immune cells, not tumor cells, are the predominant source of miR-142-3p in HNSCC. Furthermore, HNSCC single-cell expression data demonstrated that *CLIC4* is lower in tumor epithelial cells than in stromal fibroblasts and endothelial cells. Tumor-specific downregulation of *CLIC4* was confirmed in an SCC xenograft model concurrent with immune cell infiltration and miR-142-3p upregulation. These findings provide the first evidence of *CLIC4* regulation by miRNA. Furthermore, the distinct localization of CLIC4 and miR-142-3p within the HNSCC tumor milieu highlight the limitations of bulk tumor analysis and provide critical considerations for both future mechanistic studies and use of miR-142-3p as a HNSCC biomarker.

## INTRODUCTION

The chloride intracellular channel (*CLIC*) family is broadly conserved and includes six genes (*CLIC1-6*), three of which colocalize with *RUNX* and *RCAN* genes in ACD (for *AML*/*RUNX*, *CLIC*, and *DSCR*/*RCAN*) clusters (*CLIC4*, *CLIC5*, and *CLIC6* in ACD1, ACD6, and ACD21, respectively) thought to have arisen through two rounds of whole genome duplication and one segmental duplication. The maintenance of this clustering in jawed vertebrates may be due to functional cooperation during immune responses [1]. CLIC proteins are structurally metamorphic and can reversibly transit between membrane-inserted and soluble states to participate in diverse cellular functions. Membrane-inserted CLICs can form ion channels, primarily in intracellular organelles, though they are not selective for chloride ions. Several members of this protein family also exist in a soluble form, where they participate in a wide range of biochemical processes such as oxidoreduction and preventing protein dephosphorylation [2].

CLIC4 has been implicated in angiogenesis [3–5], pulmonary arterial hypertension [6, 7], epithelial differentiation [8], myofibroblast differentiation [9–11], response to oxidative stress [12–15], cellular adhesion and integrin trafficking [16–18], immunity [19–22], and cancer [23–31]. Despite the elucidation of many CLIC4 functions, little is known regarding the regulation of CLIC4 expression. Both NANOG and SOX2, but not OCT4, bind to a region approximately 2 kb upstream of the *CLIC4* transcription start site in human embryonic stem cells, but no functional studies have been performed to investigate this interaction [32]. Our laboratory identified p53 and AP-1 binding sites upstream of *CLIC4* that are required for the induction of *CLIC4* by DNA damaging stimuli and calcium-induced differentiation, respectively [8, 33, 34]. Subsequent analyses also identified MYC binding sites and that co-expression of MYC and p53 leads to synergistic activation of the *CLIC4* promoter [35]. CLIC4 expression is similarly upregulated following exposure to TNF-α and TGF-β [33, 36]. Recent studies have also shown that G-quadruplex structures near the *CLIC4* promoter are capable of regulating *CLIC4* transcription [37].

Other modulators of CLIC4 expression have also been described. In primary murine bone marrow-derived macrophages (BMDM), *Clic4* transcription is rapidly induced following treatment with lipopolysaccharide (LPS) or other toll-like receptor (TLR) agonists, even in the presence of cycloheximide, suggesting that the factors required for *Clic4* expression do not require *de novo* synthesis following TLR activation [19]. In murine fibrosarcoma cells, *Clic4* is upregulated in response to mitochondrial DNA depletion in a p53- and CREB-dependent manner [38]. In normal human bronchial epithelial cells transduced with oncogenic *KRAS*^G12V^, CLIC4 protein expression is reduced, indicating that KRAS or its downstream effectors induce negative regulators of CLIC4 expression [27]. Despite these findings, a comprehensive study of the regulatory mechanisms governing *CLIC4* expression in human cancer has never been performed.

We previously described alterations in CLIC4 expression and localization during malignant progression in several human cancer types. As tumors progress from early to late stages, detection of CLIC4 protein is lost in tumor epithelial cells with a concomitant upregulation in tumor stromal cells that acquire phenotypic markers of myofibroblasts [23]. We have shown that the stromal upregulation of CLIC4 is due to action of tumor epithelial cell-derived TGF-β on stromal fibroblasts [11]. However, the mechanism of CLIC4 loss in tumor epithelium is unknown. Here, we perform a comprehensive analysis of putative *CLIC4* regulators using genomic and epigenomic data, single-cell RNA sequencing data, molecular assays, tissue staining, and *in vivo* xenografts and show that a microRNA, miR-142-3p, is a previously undescribed regulator of *CLIC4*. We leveraged analyses with spatial resolution to demonstrate the localization and expression of both *CLIC4* and miR-142-3p within a specific cancer type, head and neck squamous cell carcinoma, which both highlights the limitations of bulk tumor analysis and introduces important considerations for the utility of CLIC4 and miR-142-3p as cancer biomarkers.

## RESULTS

### CLIC4 protein is differentially localized in human squamous carcinoma

**Figure 1:**
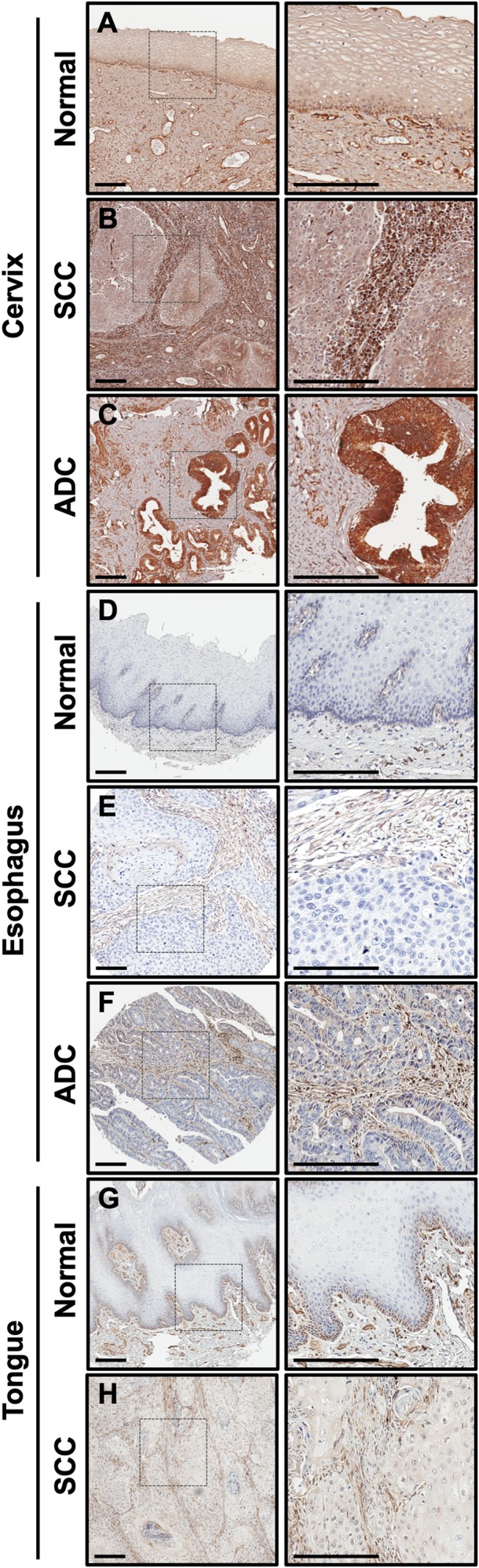
CLIC4 expression is low in epithelia of multiple squamous cancers but elevated in stromal cells. CLIC4 expression was determined by immunohistochemistry in (**A**) normal cervix, (**B**) cervical SCC, (**C**) cervical ADC, (**D**) normal esophagus, (**E**) esophageal SCC, (**F**) esophageal ADC, (**G**) normal tongue, and (**H**) tongue SCC. Left, lower magnification (10×). Dotted box represents the regions magnified on the right. Scale bar = 200 μm. SCC, squamous cell carcinoma. ADC, adenocarcinoma.

We have performed extensive immunohistochemical (IHC) staining of human tumors derived from distinct anatomical sites and cellular origins to characterize the pattern of CLIC4 protein distribution. In cancers of epithelial origin, such as squamous cell carcinoma (SCC), CLIC4 levels tend to be reduced in the epithelial compartment with a concomitant upregulation of CLIC4 protein in the nuclei and cytoplasm of tumor-associated stromal cells. However, we observed variable patterns of expression for adenocarcinomas (ADCs) derived from glandular tissue, even when assessing cancers from the same organ site. For example, in the normal stratified epithelium of the ectocervix, CLIC4 expression is highest in the basal cell layer (Figure 1A), while cervical SCCs display epithelial downregulation and stromal upregulation of CLIC4 (Figure 1B). In contrast, cervical ADCs are strongly positive for CLIC4 (Figure 1C). In the esophagus, however, both SCCs and ADCs have low CLIC4 expression in the tumor compartment and high stromal expression, while normal tissue expresses low levels of CLIC4 (Figure 1D–1F). In head and neck tissue such as tongue, CLIC4 is localized to the basal layer in normal tissue and predominant in the stroma of SCC, similar to the cervix (Figure 1G, 1H). Further heterogeneity is observed when assessing CLIC4 distribution in other tumor types (data not shown). Many factors, including cell of origin, genetic drivers, interactions with the microenvironment, and exposure to cellular stressors can promote changes in CLIC4 expression, function, and localization. The dynamic and variable regulation of CLIC4 prompted our further investigation into the mechanisms underlying these CLIC4 alterations.

### CLIC4 is rarely altered at the genomic level in human cancers

**Figure 2:**
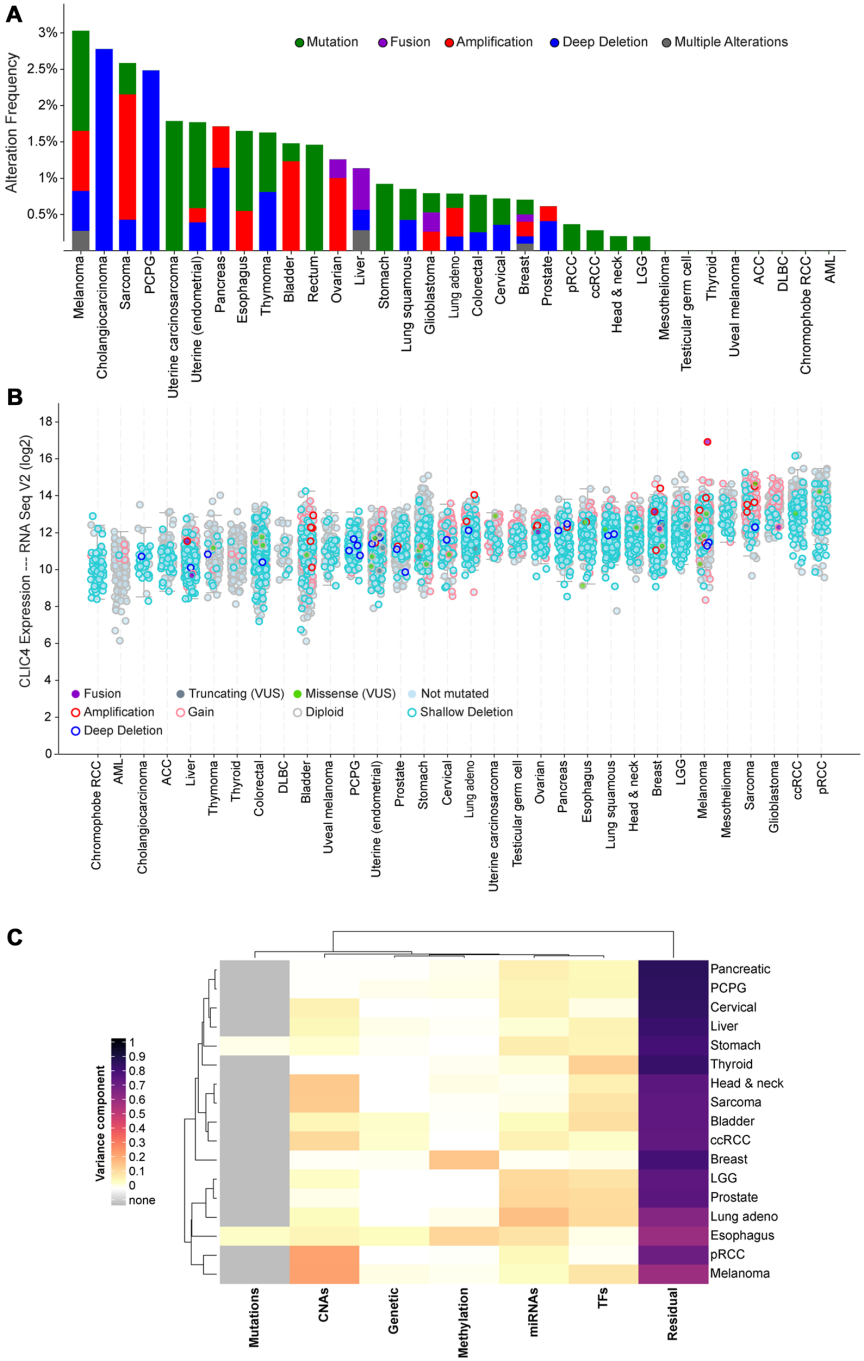
Screening for putative drivers of *CLIC4* expression variation in human cancers. (**A**) Frequency of alterations in the *CLIC4* gene in human cancers in the TCGA dataset. (**B**) Expression level of *CLIC4* RNA in human cancers in the TCGA dataset with gene alterations indicated by colors. (**C**) Variance component estimates for molecular factors on *CLIC4* expression from EDGE in TCGA. PCPG, pheochromocytoma and paraganglioma; pRCC, papillary renal cell carcinoma; ccRCC, clear cell renal cell carcinoma; LGG, brain lower grade glioma; ACC, adrenocortical carcinoma; DLBC, diffuse large B-cell lymphoma; AML, acute myeloid leukemia; VUS, variant of unknown significance; CNAs, copy number alterations; miRNAs, microRNAs; TFs, transcription factors.

We previously showed that *CLIC4* is not deleted or mutated in any of the tumor cell lines represented in the NCI-60 panel [23]. To confirm this finding in clinical specimens, we utilized cBioPortal to query datasets from the TCGA PanCancer Atlas [39, 40]. *CLIC4* was altered in 93/9870 (0.9%) of queried samples (Figure 2A). No recurrent copy number alterations or mutations predominated, and very few *CLIC4* genomic alterations were detected in each cancer type. In total, 25 amplifications, 22 homozygous deletions, 9 fusions (with *SLC45A1*, *CACHD1, UBAP2L, DNAJA4, PLOD1, CCDC28B, ARFGAP3, PACSIN2*, and *CEP85*), 4 frame shift deletions (recurrent, K204Nfs*11), 29 missense mutations (none recurrent), 3 nonsense mutations (S132*, E239*, and E213*), and 1 nonstop mutation (*254Yext*26) were present (Supplementary Table 1). Two samples had both fusions and amplifications. The missense mutations are of unknown significance, but two have the potential to disrupt phosphorylation sites that we previously identified (S27N, putative CK2 site; S38F, putative PKC site) [33]. When assessing *CLIC4* RNA expression levels across these datasets, no trends were observed regarding expression and cell/tissue type or the presence of genomic alterations (Figure 2B). Thus, differential regulation of *CLIC4* in cancer must be due to transcriptional/translational or post-transcriptional/translational mechanisms.

### Putative transcriptional regulators of CLIC4 expression

To further assess putative regulators of *CLIC4* expression other than genomic alteration, we utilized the Exploring Drivers of Gene Expression in The Cancer Genome Atlas (EDGE in TCGA) application. EDGE in TCGA utilizes processed TCGA level 3 data to attribute the variance in gene expression to several molecular variables such as somatic mutations, germ-line polymorphisms, promoter methylation, or miRNA and transcription factor abundance [41]. No major source of molecular variation was apparent across the cancer types, exemplified by the predominance of the residual component (Figure 2C). This analysis was limited to samples present in the TCGA for which all data types were available (*n* = 3228).

**Figure 3:**
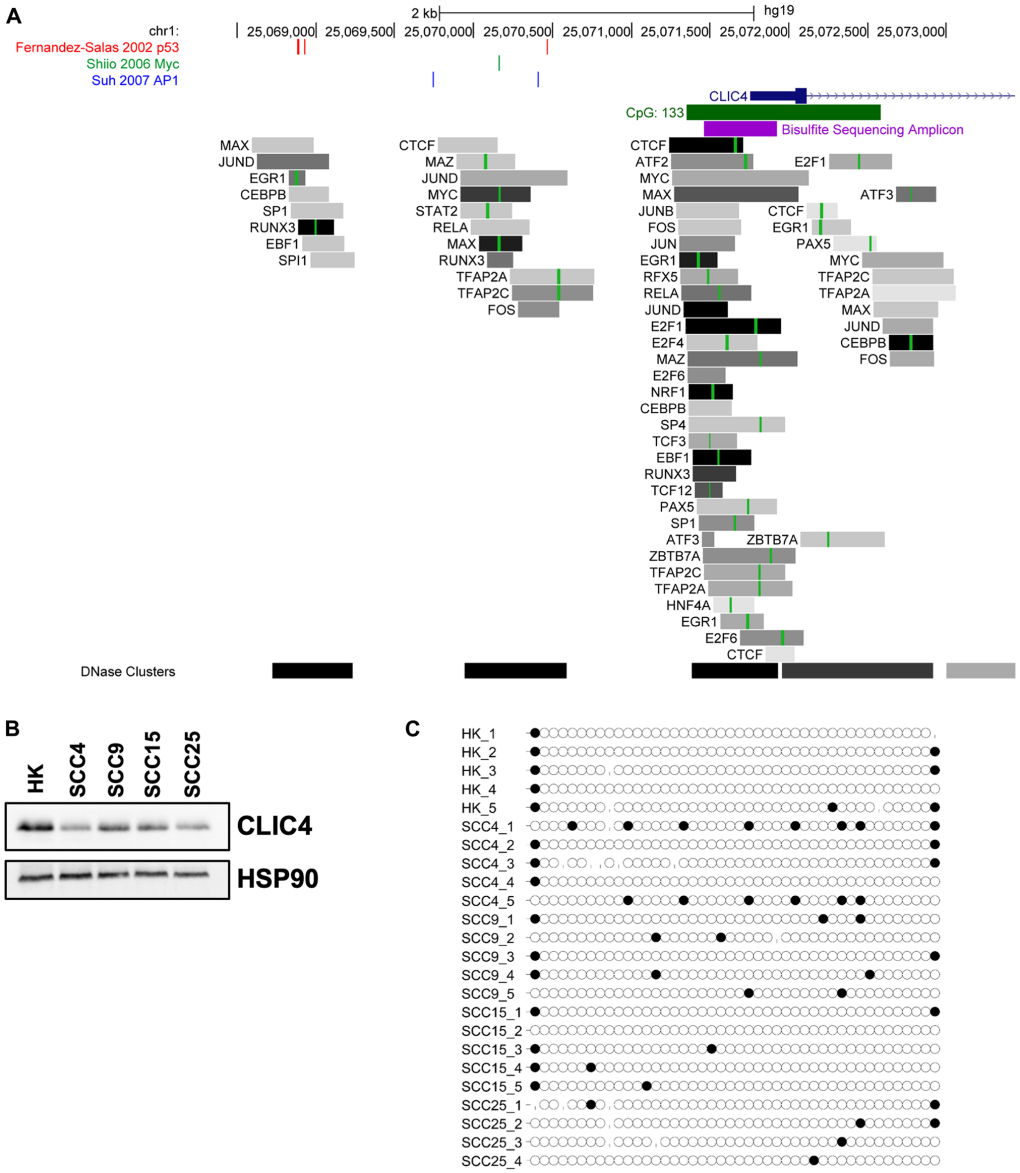
Cancer-associated transcription factors, but not CpG island methylation, regulate *CLIC4* expression. (**A**) Schematic of the promoter region of the human *CLIC4* locus from the UCSC Genome Browser. The location of previously published p53 binding sites are shown in red, Myc binding sites in green, and AP1 binding sites in blue. The promoter-associated CpG island is indicated with a green rectangle, and region amplified for bisulfite sequencing with a purple rectangle. Tracks from ENCODE transcription factor ChIP-seq are also shown. Each gray box represents the peak of transcription factor occupancy, and the shading intensity is proportional to the signal strength. Green lines within the box represent canonical binding motifs for the transcription factor. (**B**) Western blot analysis of CLIC4 and HSP90 (loading control) protein expression in cultures of primary keratinocytes (HK) and SCC4, SCC9, SCC15, and SCC25 cell lines. (**C**) Bisulfite sequencing reads from the bisulfite sequencing amplicon indicated in (A) from DNA extracted from the cells shown in (B). Each circle represents a cytosine residue from a CpG dinucleotide, with 44 assayed within the amplicon. Black filled circles represent a methylated residue. Missing circles indicate incomplete sequencing data. Reads from 4–5 independent sequencing clones are shown.

Additional information about transcription factor binding and promoter methylation was obtained using the UCSC Genome Browser and ENCODE Transcription Factor ChIP tracks [42]. There are three major DNase hypersensitivity (DHS) clusters spanning and upstream of the *CLIC4* promoter where transcription factor binding is localized (Figure 3A). Transcription factor binding is most enriched at the promoter-proximal DHS cluster, with binding of factors such as ATF2 and 3, MYC and MAX, FOS and JUN, NRF1, CEBPB, TCF3, EBF1, and RUNX3 that are known to regulate immune cell function and response to stress. This list is not exhaustive, as it is limited to the 161 transcription factors in 91 cell lines represented within the ENCODE dataset. The second DHS cluster co-localizes with the p53, MYC, and AP-1 sites and the third DHS cluster co-localizes with the distal p53 sites we previously described [8, 34, 35]. Differential expression and activity of these upstream regulators are likely to contribute in part to changes in *CLIC4* expression.

A CpG island spans the *CLIC4* promoter region, so we performed bisulfite sequencing of normal human keratinocytes and several squamous cell carcinoma cell lines with differential CLIC4 expression (Figure 3B) to determine if CpG methylation could account for these changes. An amplicon containing 50 of the 133 CpGs within the island was amplified from bisulfite-converted DNA, cloned, and sequenced. No substantial differential methylation was detected (Figure 3C). Queries of publicly available data also showed limited methylation in this region (data not shown), suggesting that promoter methylation is not a mechanism of *CLIC4* loss in epithelial tumors.

### CLIC4 expression can be modulated by miRNAs

**Figure 4:**
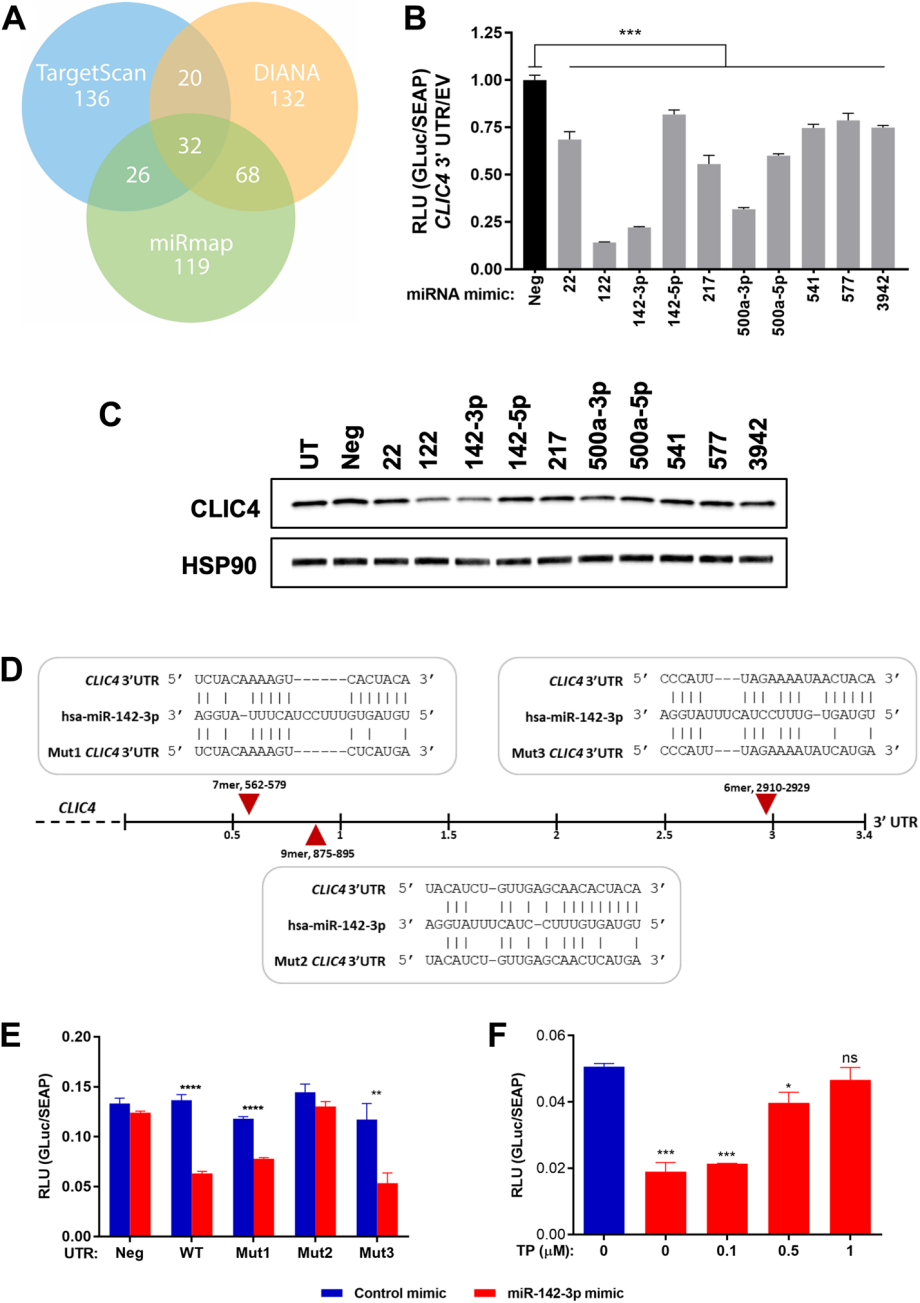
miR-142-3p targets *CLIC4* by recognizing a specific site in the *CLIC4* 3′UTR. (**A**) Number of putative miRNAs proposed to target *CLIC4* using the TargetScan (blue), DIANA (orange), or miRmap (green) search algorithms. The numbers of overlapping miRNAs are indicated at the intersection of each circle. (**B**) Relative luminescence detected at 48 hours following the co-transfection of either *Gaussia* luciferase (GLuc) empty vector (EV) or *CLIC4* 3′UTR-GLuc and each miRNA mimic (20 nM) in 293T cells. ^***^ *p* ≤ 0.001 for ANOVA with Dunnett’s correction for multiple comparisons, Neg vs. each mimic. (**C**) Western blot analysis of CLIC4 and HSP90 (loading control) protein expression at 48 hours after transfection of each indicated miRNA mimic (20 nM) in 293T cells. (**D**) Schematic of the *CLIC4* 3′UTR with the sequence and relative position of each putative miR-142-3p binding site indicated with a red arrow. The disruption of base pairing in the miRNA seed region for each mutagenized reporter plasmid (Mut1-3) is shown below each site. (**E**) Relative luminescence detected at 48 hours following the co-transfection of either *Gaussia* luciferase (GLuc) empty vector (Neg), wild-type *CLIC4* 3′UTR-GLuc (WT), or Mut1-3 *CLIC4* 3′UTR-GLuc (Mut1, Mut2, Mut3) and either non-targeting control mimic or miR-142-3p mimic (10 nM) in 293T cells. ^**^ *p* ≤ 0.01, ^****^ *p* ≤ 0.0001 for Student’s *t*-test comparing control mimic vs. miR-142-3p mimic for each UTR vector. (**F**) Relative luminescence detected at 72 hours following the co-transfection of wild-type *CLIC4* 3′UTR-GLuc, non-targeting control mimic or miR-142-3p mimic (10 nM) and increasing concentrations of target protector (TP) designed to block the interaction of miR-142-3p with site 2 of the *CLIC4* 3′UTR in 293T cells. ^*^ *p* ≤ 0.05, ^***^ *p* ≤ 0.001, ns *p* > 0.05 for ANOVA with Dunnett’s correction for multiple comparisons, control mimic vs. each miR-142-3p/TP concentration. For (B), (E), and (F), secreted alkaline phosphatase (SEAP) is expressed from an independent promoter in the sample plasmid and was used to normalize for transfection efficiency. UTR, untranslated region; RLU, relative luminescence units; Neg, negative control (non-targeting) mimic; UT, untransfected.

Because miRNAs are heavily dysregulated in cancer and can promote tumor growth by targeting factors involved in apoptosis [43], one of many functions we have defined for CLIC4 [44], we sought to identify miRNAs that can modulate CLIC4 expression. To computationally predict miRNAs targeting *CLIC4*, we queried TargetScan [45], DIANA-microT [46], and miRmap [47], the methodologies with the best predictive performance among all currently available tools [48]. Most of the putative targeting miRNAs were identified by only one algorithm (TargetScan, 136; DIANA, 132; miRmap, 119), so we chose to first investigate the 32 miRNAs shared by all three lists (Figure 4A, Supplementary Table 2). We further ranked the miRNAs by their algorithm scores and pursued the ten with the best rank sum. To test their ability to regulate CLIC4 expression, we performed an *in vitro* reporter assay by co-transfecting a plasmid containing the *CLIC4* 3′UTR downstream of luciferase and mimics for each miRNA into 293T cells. All ten mimics reduced luciferase expression as compared to a non-targeting negative control mimic, but to different extents (Figure 4B). miR-122 and miR-142-3p induced the strongest repression, which was validated at the CLIC4 protein level in 293T cells (Figure 4C). miR-122 is largely considered liver-specific and has been implicated in hepatocellular carcinoma [49], while miR-142-3p is highly expressed in hematopoietic cells and has been investigated in multiple cellular contexts and diseases [50]. Therefore, we chose to focus on miR-142-3p for subsequent experiments.

### miR-142-3p represses CLIC4 by interacting with the CLIC4 3′UTR

Several putative miR-142-3p binding sites were identified in the *CLIC4* 3′UTR, a 7-mer at position 562-579, a 9-mer at position 875-895, and a 6-mer at position 2910-2929. Key residues in the seed binding region of each site of the *CLIC4* 3′UTR reporter plasmid were mutagenized individually to disrupt the ability of miR-142-3p to bind (Figure 4D). Only mutagenesis of the second putative site (9-mer at 875-895 bp downstream of *CLIC4* stop codon) was able to abolish the repression, indicating that this site is required for miR-142-3p to exert its repressive effect on CLIC4 (Figure 4E). This was further validated with a target protector (TP), a modified RNA designed to bind to and disrupt this specific site in the *CLIC4* 3′UTR without affecting the stability of the mRNA. Co-transfection of the wild-type *CLIC4* 3′UTR reporter plasmid, miR-142-3p mimic, and increasing concentrations of the TP attenuated miR-142-3p-induced repression in a dose-dependent manner (Figure 4F). Thus, *CLIC4* is a bona fide target of miR-142-3p, which targets the transcript through interaction with a site at position 875 of the *CLIC4* 3′UTR.

### miR-142-3p can target CLIC4 in HNSCC

**Figure 5:**
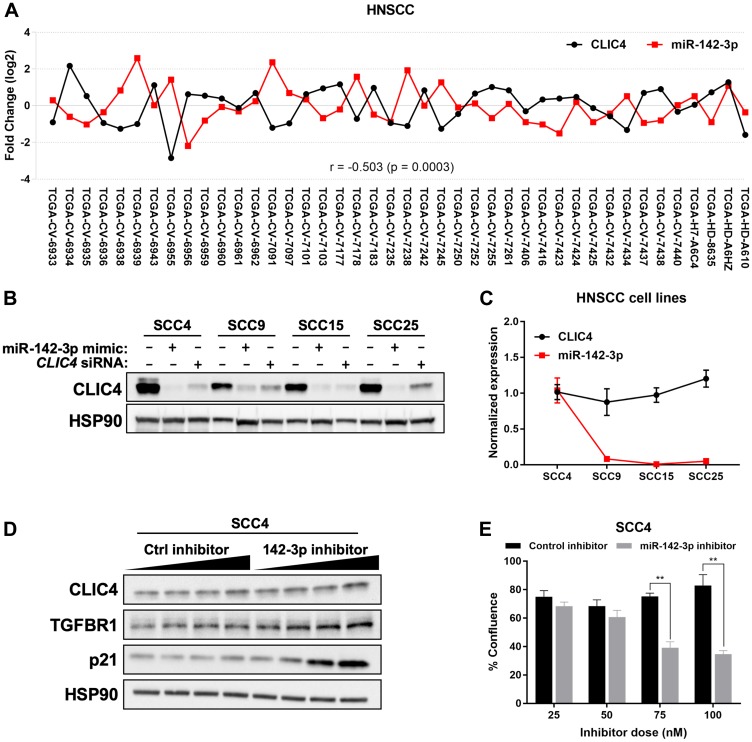
miR-142-3p modulates *CLIC4* expression in HNSCC. (**A**) Fold change in *CLIC4* and miR-142-3p in tumor compared with matched normal tissue for HNSCC from TCGA data processed by miRTarBase (*n* = 42). Statistics represent the Pearson correlation coefficient. Individual TCGA identifiers are shown on the x-axis. (**B**) Western blot analysis of CLIC4 and HSP90 (loading control) protein expression at 72 hours after transfection of miR-142-3p mimic or *CLIC4* siRNA (20 nM) in SCC4, SCC9, SCC15, and SCC25 cell lines. (**C**) qPCR analysis of endogenous *CLIC4* and miR-142-3p RNA expression in SCC4, SCC9, SCC15, and SCC25 cell lines. mRNA expression was normalized using the expression of *RPL37*, and miRNA expression was normalized using the expression of *RNU6*. (**D**) Western blot analysis of CLIC4, TGFBR1, p21, and HSP90 (loading control) protein expression at 48 hours after transfection of 25, 50, 75, or 100 nM (increasing dose indicated by black wedge) of control inhibitor or miR-142-3p inhibitor in SCC4 cells. (**E**) Percent confluence of SCC4 cells in plates from (D) at 48 hours post-transfection. Black, control inhibitor; Gray, miR-142-3p inhibitor. ^**^ *p* ≤ 0.01 for Student’s *t*-test comparing control inhibitor vs. miR-142-3p inhibitor at each individual dose. HNSCC, head and neck squamous cell carcinoma; TCGA, The Cancer Genome Atlas; qPCR, quantitative polymerase chain reaction.

Upregulation of miR-142-3p in head and neck squamous cell carcinoma (HNSCC) has been reported previously [51]. To determine the relationship between miR-142-3p and *CLIC4* in HNSCC, we queried miRTarBase, which utilizes a selection of curated miRNA-seq and RNA-seq data from TCGA that contain matched tumor and normal samples from the same patients [52]. For HNSCC (*n* = 42), miR-142-3p and *CLIC4* displayed an inverse expression pattern with a significant negative Pearson correlation (r = –0.503, *p* = 0.0003; Figure 5A). We surveyed several HNSCC cell lines (SCC4, 9, 15, and 25) and found that while CLIC4 is moderately abundant *in vitro*, its protein level can be downregulated by the addition of miR-142-3p mimic, to a level comparable or greater than that of a *CLIC4* siRNA (Figure 5B). Of these cell lines, only SCC4 expressed modest levels of endogenous miR-142-3p, but this did not correlate with the level of *CLIC4* (Figure 5C). Following the addition of a miR-142-3p inhibitor RNA in SCC4 cells, we observed a slight dose-dependent increase in the protein level of CLIC4 and transforming growth factor beta receptor 1 (TGFBR1), an experimentally validated miR-142-3p target [53] (Figure 5D). We also observed that cultures treated with 75–100 nM miR-142-3p inhibitor failed to become confluent (Figure 5E), potentially due to upregulation of CLIC4 or p21 (Figure 4D) and subsequent cell cycle arrest or apoptosis [44, 54]. These data demonstrate that exposure to exogenous miR-142-3p is capable of downregulating CLIC4 protein in human HNSCC cell lines and endogenous miR-142-3p expression has a significant reciprocal relationship with *CLIC4* expression in HNSCC tissue *in vivo*.

### miR-142-3p and CLIC4 expression levels are inversely correlated in progressive squamous cancers

**Figure 6:**
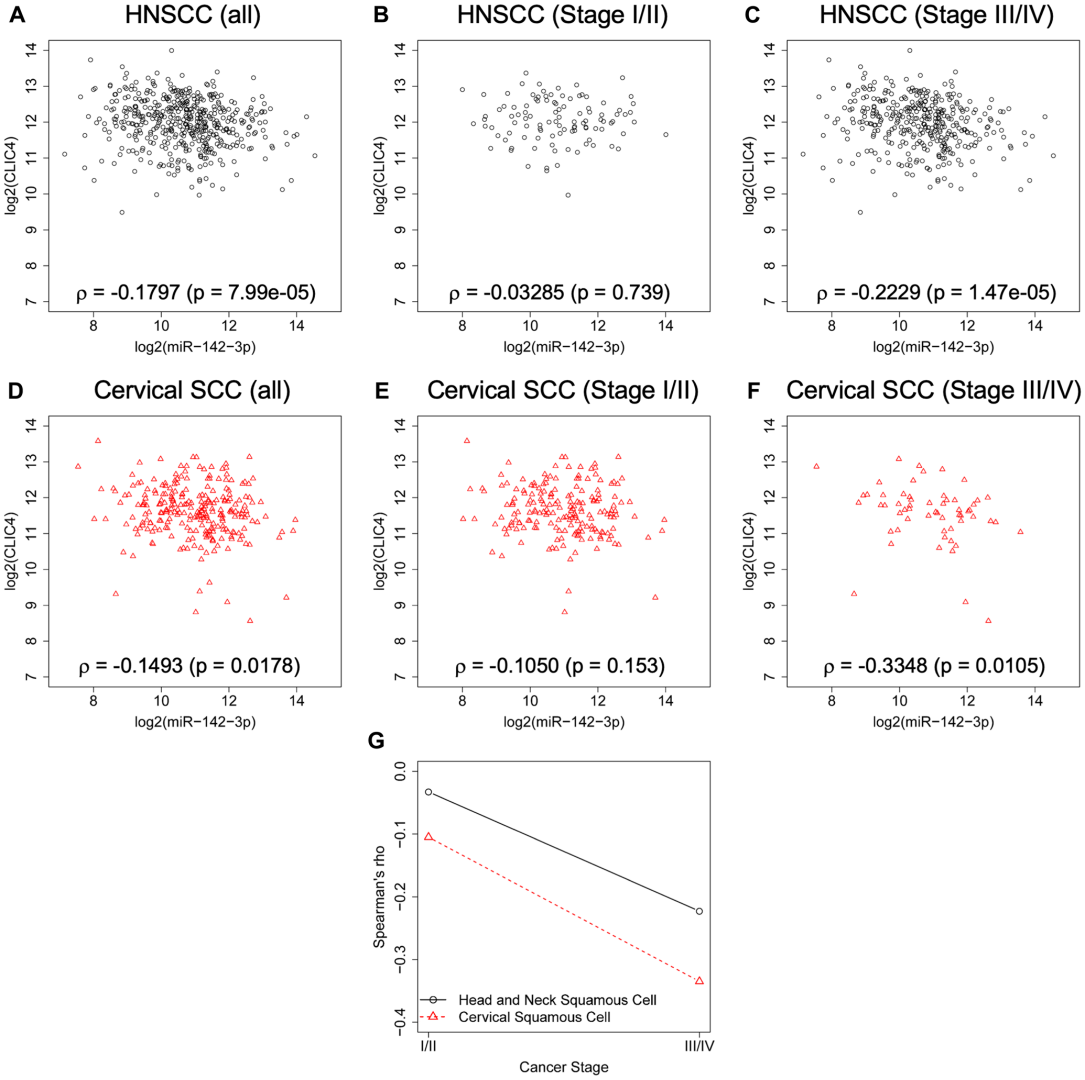
*CLIC4* and miR-142-3p are inversely related in advanced squamous cell cancers. Relationship between the expression level of *CLIC4* and miR-142-3p in subsets of TCGA data. (**A**) Expression in all primary HNSCC tumors with complete mRNA and miRNA data (*n* = 475). (**B**) Expression in stage I (*n* = 26) and stage II (*n* = 78) HNSCC tumors from (A) (*n* = 104). (**C**) Expression in stage III (*n* = 86) and stage IV (*n* = 285) HNSCC tumors from (A) (*n* = 371). (**D**) Expression in all primary cervical SCC tumors with complete mRNA and miRNA data (*n* = 252). (**E**) Expression in stage I (*n* = 125) and stage II (*n* = 62) cervical SCC tumors from (D) (*n* = 187). (**F**) Expression in stage III (*n* = 42) and stage IV (*n* = 16) cervical SCC tumors from (D) (*n* = 58). Tumors with no staging data were excluded from the staged analysis (*n* = 7). Statistics represent Spearman’s rho. (**G**) Comparison of the value of Spearman’s rho for stage I/II and stage III/IV tumors. HNSCC, black open circles; Cervical SCC, red open triangles; HNSCC, head and neck squamous cell carcinoma; SCC, squamous cell carcinoma.

miRTarBase miRNA/mRNA TCGA expression data (Figure 5A) was limited to samples with matched normal tissue (*n* = 42). To determine if the inverse relationship between the expression of miR-142-3p and *CLIC4* is preserved when considering all tumor samples, we compared their expression values in the entire TCGA HNSCC dataset (*n* = 475). We also assessed cervical SCC (*n* = 252), for which we observed epithelial downregulation of CLIC4 (Figure 1B). In HNSCC, there was a significant negative correlation between *CLIC4* and miR-142-3p expression (Figure 6A, ρ = –0.1797, *p* = 7.99e-05). This relationship was not significant in stage I/II cancers (Figure 6B, ρ = –0.03285, *p* = 0.739), but there was a stronger negative correlation in stage III/IV cancers (Figure 6C, ρ = –0.2229, *p* = 1.47e-05). The same held true for cervical SCC, where a negative correlation was observed (Figure 6D, ρ = –0.1493, *p* = 0.0178), and while the correlation was not significant for stage I/II cancers (Figure 6E, ρ = –0.1050, *p* = 0.153), a stronger negative correlation was observed for stage III/IV cervical SCC (Figure 6F, ρ = –0.3348, *p* = 0.0105). The more notable inverse relationship between *CLIC4* and miR-142-3p in stage III/IV squamous cancers (Figure 6G) suggests that miR-142-3p regulation of *CLIC4* may occur predominantly in advanced cancers.

### scRNA-seq reveals abundant CLIC4 expression in HNSCC tumor stroma

**Figure 7:**
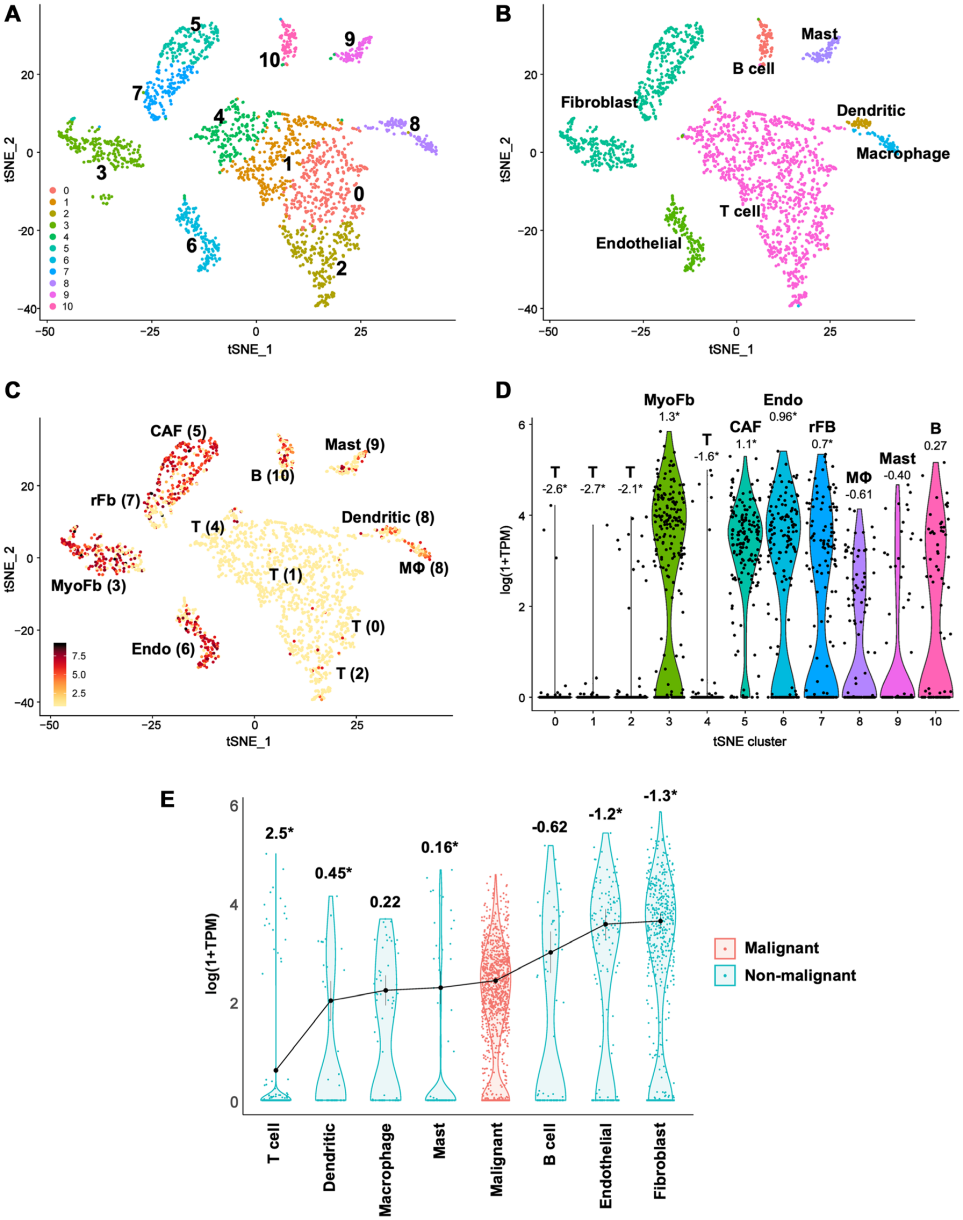
Single-cell RNA-seq data reveal lower *CLIC4* expression in malignant tumor cells than in stromal fibroblasts in HNSCC. (**A–C**) t-distributed Stochastic Neighbor Embedding (t-SNE) plots. (A) t-SNE clusters of all non-malignant cell types. Ten unique clusters were discriminated. (B) Cell type annotation from Puram et al. [57]. (C) *CLIC4* expression within t-SNE clusters, imaged values are log (1+TPM). Additional cell type annotations from Puram et al. [57] are indicated near the relevant cluster. (**D**) Violin plots of *CLIC4* expression distribution in cells assigned to the t-SNE clusters. Numbers shown are average log fold change in *CLIC4* expression within a cluster vs. the other populations of cells, ^*^indicates significant differential expression detected in a cluster (Bonferroni corrected *p* ≤ 0.05). (**E**) Violin plots of *CLIC4* expression distribution in the identified cell types. The distributions are ordered by the mean expression of *CLIC4* within a cell type. Numbers shown are average log fold change in *CLIC4* expression within malignant cells vs. the specified non-malignant cell type, ^*^indicates significant differential expression between malignant and non-malignant cells (Bonferroni corrected *p* ≤ 0.05). B, B cell; CAF, cancer-associated fibroblast; Endo, endothelial; MΦ, macrophage; MyoFb, myofibroblast; rFb, resting fibroblast; T, T cell; TPM, transcripts per kilobase million.

Though we observed significant negative correlations between *CLIC4* and mir-142-3p expression in human HNSCC (Figure 6), the modest amplitude of the correlation in light of strong molecular *in vitro* data (Figure 4, Figure 5B) led us to question whether the strength of the interaction was masked by the loss of spatial resolution in TCGA bulk analysis and disparate levels of CLIC4 expression between tumor and stromal compartments [23]. Cellular deconvolution techniques can be applied to bulk tumor gene expression datasets to estimate tumor purity and the infiltration of stromal and immune cells [55], but recent advances in single-cell RNA-sequencing (scRNA-seq) allow for direct profiling of intratumoral cellular heterogeneity and expression variation in discrete compartments [56]. We assessed *CLIC4* expression in the scRNA-seq dataset generated by Puram et al. [57], which profiled 6,000 single cells from HNSCC patients. We were unable to leverage this dataset to compare the levels of *CLIC4* and miR-142-3p in single cells because current technical challenges limit simultaneous detection of miRNA and mRNA transcriptomes [58]. The curated data distinguished malignant from non-malignant cells based on global expression patterns, epithelial origin, and inferred karyotype. Our application of dimensional reduction to the non-malignant cell data revealed ten distinct clusters based on expression states (Figure 7A, Supplementary Table 3) that broadly match the cell type annotations distinguished by Puram et al. (Figure 7B). *CLIC4* is highly expressed in HNSCC tumor-associated fibroblasts and endothelial cells, expressed at lower levels in macrophages, mast cells, and B cells, and is remarkably absent in T cells (Figure 7C and 7D, Supplementary Table 3). Among fibroblast subsets, *CLIC4* is highest in myofibroblasts, which is consistent with our previous findings that CLIC4 plays an integral role in TGF-β-dependent myofibroblast differentiation [11]. *CLIC4* is also significantly higher in cancer-associated fibroblasts (CAFs) than in other non-malignant cell types, and is among the top 150 differentially expressed genes that differentiate the CAF1 from CAF2 subsets as described by Puram et al. [57]. The abundance of *CLIC4* in stromal cells prompted us to further compare the relative expression level of *CLIC4* between the malignant tumor cell population and each non-malignant cell type (Figure 7E, Supplementary Table 3). *CLIC4* expression was significantly lower in the malignant cells than in tumor-associated fibroblasts (-3.6-FC; -1.3-logFC) and endothelial cells (-3.3-FC; -1.2-logFC). These findings suggest that the contribution of stromal *CLIC4* can confound analyses regarding the level of *CLIC4* in tumors and the miR-142-3p/*CLIC4* relationship in bulk expression data, necessitating analyses that preserve spatial localization.

### Stromal immune cells express miR-142-3p in HNSCC tissue

**Figure 8:**
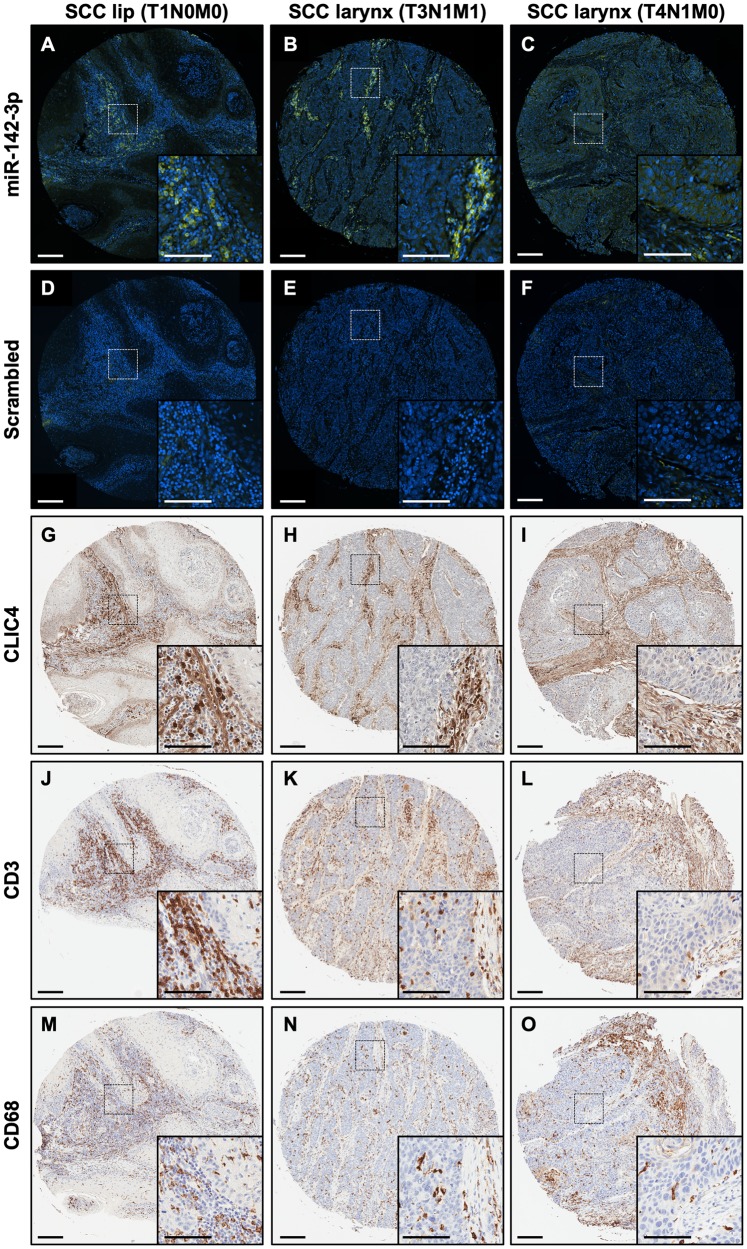
miR-142-3p is expressed in the stromal compartment along with immune cells in HNSCC. (**A**–**C**) *In situ* hybridization with a miR-142-3p probe (green) in human HNSCC. Nuclei are counterstained with DAPI. (**D**–**F**) *In situ* hybridization with a scrambled probe (green) in human HNSCC. Nuclei are counterstained with DAPI. (**G**–**I**) Immunohistochemistry for CLIC4 in human HNSCC. (**J**–**L**) Immunohistochemistry for CD3, a marker of T cells, in human HNSCC. (**M**–**O**) Immunohistochemistry for CD68, a marker of monocytes, in human SCC. Each panel shows a lower magnification (10×) and a dotted box shows the region that is magnified in the inset. Primary scale bar = 200 μm. Inset scale bar = 100 μm. The first column shows consecutive sections of lip SCC tissue (stage T1N0M0), the second column shows consecutive sections of larynx SCC (stage T3N1M1), and the third column shows consecutive sections of larynx SCC (stage T4N1M0). HNSCC, head and neck squamous cell carcinoma; SCC, squamous cell carcinoma.

To address the compartmentalization of CLIC4- and miR-142-3p-expressing cells within tumors, we performed *in situ* hybridization (ISH) for miR-142-3p on serial sections of a human HNSCC tumor tissue microarray. Bright punctate positive signals for the miR-142-3p probe were abundant in the tumor stroma (Figure 8A–8C), while only signals from auto-fluorescing red blood cells were observed for the control scrambled probe (Figure 8D–8F). Subsequent immunohistochemical staining of a serial section for CLIC4 confirmed lower expression in tumor epithelia relative to the stromal compartment regardless of location or stage (Figure 8G–8I). Further staining for T cells (CD3; Figure 8J–8L) and macrophages (CD68; Figure 8M–8O) in additional serial sections found that these immune cells were also abundant in the tumor stroma concurrent with stromal miR-142-3p-positive cells. Although co-localization could not be definitively determined with the techniques employed here, extensive literature describing roles for miR-142-3p within hematopoietic cells strengthens the likelihood that miR-142-3p is present within tumor-associated stromal immune cells [50]. Diffuse miR-142-3p staining was detected in the tumor compartment in some but not all tumors (Figure 8C), suggesting heterogeneity in tumor epithelial cell expression of miR-142-3p or paracrine uptake from neighboring immune cells, which has been documented [59]. Nevertheless, CLIC4 tissue expression appears to be independent of the extent of miR-142-3p-positive infiltrating cells, indicating that additional factors contribute to CLIC4 regulation *in vivo*.

### CLIC4 expression is reduced and miR-142-3p expression elevated when SCC cell lines are transferred from *in vitro* to form tumors *in vivo*

**Figure 9:**
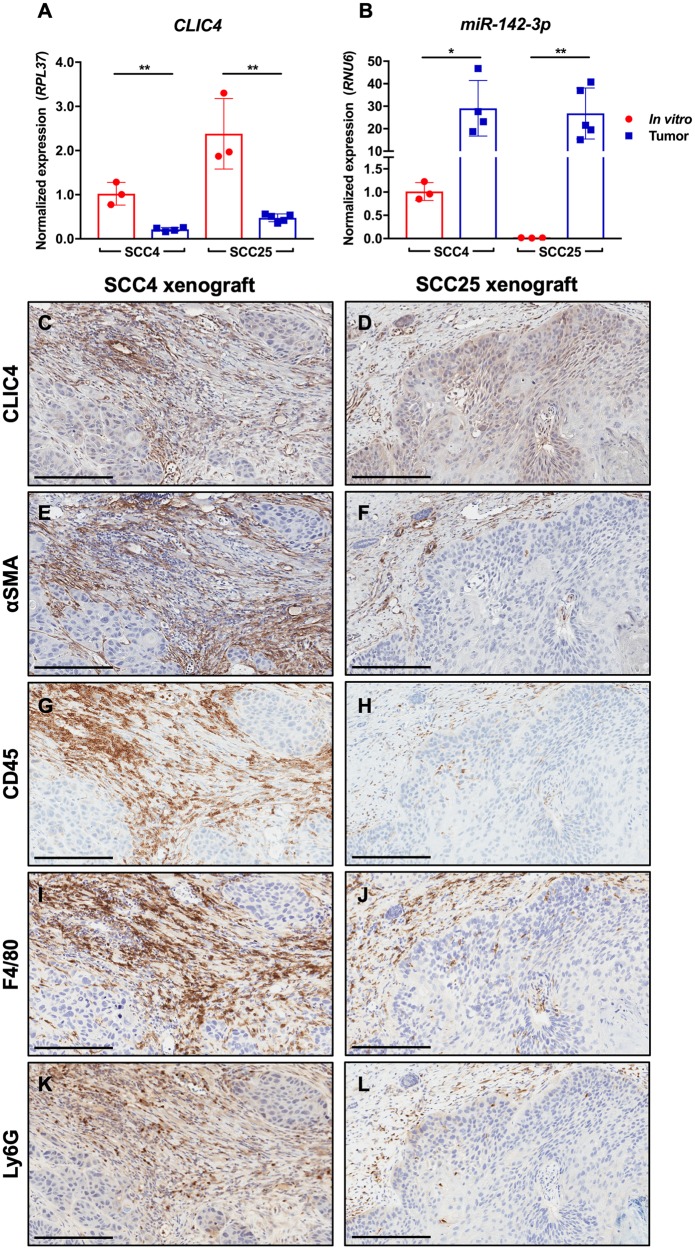
*CLIC4* is downregulated in SCC xenografts concurrent with immune cell infiltration and miR-142-3p upregulation. SCC cell line xenografts contain more miR-142-3p and less CLIC4 *in vivo*. (**A**) qPCR analysis of human *CLIC4* RNA expression in SCC4 and SCC25 cells cultured *in vitro* and from *in vivo* xenograft tumors in nude mice. Human-specific primers differentiate between SCC-derived and host-derived (mouse) *CLIC4*. ^**^ *p* ≤ 0.01 for Student’s *t*-test comparing *in vitro* vs. tumor gene expression for SCC4 or SCC25. (**B**) qPCR analysis of miR-142-3p expression in samples from (A). Human- and mouse-derived miR-142-3p cannot be distinguished. ^*^ *p* ≤ 0.05, ^**^ *p* ≤ 0.01 for Student’s *t*-test comparing *in vitro* vs. tumor gene expression for SCC4 or SCC25. (**C**–**D**) Immunohistochemistry for CLIC4. (**E**–**F**) Immunohistochemistry for αSMA, a marker of activated fibroblasts. (**G**–**H**) Immunohistochemistry for CD45, a marker of mouse hematopoietic cells. (**I**–**J**) Immunohistochemistry for F4/80, a marker of mouse macrophages. (**K**–**L**) Immunohistochemistry for Ly6G, a marker of mouse granulocytes/neutrophils. For all immunohistochemistry, the left column shows consecutive sections of a SCC4 xenograft, and the right column shows consecutive sections of a SCC25 xenograft. Magnification 10 ×. Scale bar = 200 μm. qPCR, quantitative polymerase chain reaction; αSMA, alpha smooth muscle actin.

Based on the abundance of miR-142-3p in human HNSCC stroma, we reasoned that *CLIC4* may be differentially regulated *in vivo* as compared with *in vitro* culture because of exposure to microenvironmental factors such as miR-142-3p. We generated xenografts in nude mice with two HNSCC cell lines, SCC4, which expresses low levels of endogenous miR-142-3p, and SCC25, which does not express miR-142-3p *in vitro* (Figure 5C). Tumor-specific *CLIC4* expression was measured using qPCR primers that recognize human (tumor), but not mouse (host), *CLIC4*. However, because the sequences of human and mouse miR-142-3p are identical, the source of miR-142-3p *in vivo* could not be distinguished using qPCR. Both SCC4 and SCC25 expressed lower levels of *CLIC4* when placed *in vivo* than when grown in culture (Figure 9A). Conversely, miR-142-3p was more highly expressed in bulk tumors than in either isolated cell line *in vitro* (Figure 9B). Immunohistochemical staining showed that CLIC4 expression was lower in the tumor compartment in both SCC4 and SCC25 xenografts than in the tumor stroma, which was also positive for αSMA, a marker of myofibroblasts (Figure 9C–9F). Furthermore, despite the use of nude mice that are deficient for T cells, tumors showed infiltration of cells expressing CD45 (Figure 9G, 9H), which is expressed on all nucleated hematopoietic cells, including F4/80-positive macrophages (Figure 9I, 9J) and Ly6G-positive granulocytes/neutrophils (Figure 9K, 9L). While these data do not eliminate the possibility that the SCC cell lines upregulated miR-142-3p when placed *in vivo*, the abundance of infiltrating immune cells make it likely that the apparent upregulation of miR-142-3p is due to the presence of these high miR-142-3p expressors in bulk tumor lysates. Furthermore, the ability to differentiate between tumor and host *CLIC4* with species-specific primers circumvents the limitations of bulk analysis and confirms downregulation of *CLIC4* following the transition from *in vitro* to *in vivo* growth and exposure to the microenvironmental milieu.

## DISCUSSION

The reduction of CLIC4 in tumor cells in progressing cancers of certain organs, particularly squamous cancers, suggests a tumor suppressor role for the protein. Indeed, in a cutaneous cancer model, overexpression of *CLIC4* in epidermis by transgene targeting or administration of exogenous *CLIC4* via viral transduction are effective in reducing tumor growth [24]. We now show three tumor types (cervix, esophagus, and head and neck) with low CLIC4 expression in parenchyma relative to stroma. To elucidate the molecular basis for this phenomenon, we first assessed TCGA data and identified few overall and no recurrent *CLIC4* mutations and no predominant molecular variable that could explain the changes in *CLIC4* expression. We used ENCODE data to analyze protein binding near the *CLIC4* promoter and identified binding by members of transcription factor families with known involvement in cancer such as MYC (MYC, MAX), p53 (TP53), AP-1 (FOS, JUN, JUNB, JUND, ATF2), NF-κB (RELA), CTCF, PAX5, and RUNX3 [60]. Although there is a CpG island spanning the *CLIC4* promoter, we did not detect differential methylation of this region, suggesting that *CLIC4* is not epigenetically silenced by DNA methylation in this context. We also demonstrated for the first time that several miRNAs, including miR-142-3p, are capable of downregulating *CLIC4* at the post-transcriptional level. These data lead us to propose that deregulation of *CLIC4* in cancer results from the convergence of multiple upstream and downstream regulatory factors, and that different mechanisms of regulation are likely in play within different cell types within the tumor and microenvironment.

miRNAs are heavily dysregulated in cancer, and a single miRNA may have specificity for several hundred mRNA targets [61]. Consequently, aberrant miRNA expression can promote broad regulatory changes that sustain malignant transformation or maintenance [43]. Divergent roles for miR-142-3p as both an oncogene and a tumor suppressor are pervasive in the literature [50], though it is not clear if this is due to cancer-specific intrinsic dependencies or experimental artifacts. For instance, transfection with miRNA mimics at 100 nM results in an intracellular mimic level of 1.8 million copies after 6 hours, a concentration in vast excess of the 100,000 copies of total endogenous mature miRNA per cell. Exposure to supraphysiological miRNA levels leads to non-specific gene expression changes through several mechanisms [62]. Variation in response across cell lines can also be ascribed to differential sensitivity to double-stranded RNA exposure and non-specific interferon-dependent apoptosis [63]. In our study, we sought to limit the use of miRNA mimics and used them only for a few confirmatory studies. We also used mutagenesis to definitively demonstrate that miR-142-3p-mediated downregulation of *CLIC4* is dependent on a specific site in the *CLIC4* 3′UTR. Most importantly, we relied on endogenous miR-142-3p whenever possible and employed *in vivo* characterization to identify putative interactions between *CLIC4* and miR-142-3p in biologically relevant contexts.

We chose to focus on miR-142-3p because of its documented roles in cancer [50], but had difficulty identifying HNSCC cell lines with endogenous expression of miR-142-3p. SCC4 cells had detectable levels of expression, but neither the additional UM-SCC cell lines [64] we screened nor *in silico* analysis of the Cancer Cell Line Encyclopedia (CCLE) [65] identified any other HNSCC cell lines with endogenous miR-142-3p expression. In fact, CCLE data suggest that miR-142-3p expression is largely restricted to hematopoietic cells and its genomic locus is heavily methylated in most other cell types (data not shown). Indeed, treatment with 5-aza-2’-deoxycytidine to inhibit CpG methylation is sufficient to induce miR-142-3p expression in murine fibroblasts and human mesenchymal cells, suggesting that the *miR-142* gene is epigenetically repressed by DNA methylation in non-hematopoietic cell types [66, 67]. Another study suggests that miR-142-3p is expressed in oral SCC cell lines but selectively secreted in extracellular vesicles (EVs) to decrease its intracellular concentration [68], but we could not independently replicate these findings and CCLE data indicate that the *miR-142* locus is also methylated in the Cal27 cell line used in the study (data not shown). Nonetheless, other data confirm that miR-142-3p is indeed released in EVs, particularly from bone marrow-derived cells, and can affect target gene expression in recipient cells in a paracrine manner. This has been directly documented for macrophages [59, 69, 70], T cells [71–73], and bone marrow-derived mesenchymal stem/stromal cells [74], with uptake and activity in recipient endothelial and cancer cells [59, 69–71, 74]. Additional data support the use of miR-142-3p as a biomarker due to elevation of circulating miR-142-3p levels in various pathological states [75–96]. In fact, a recent review defined miR-142-3p as one of a core set of biomarker miRNAs for atopic diseases because of its frequent association with allergic inflammation and immune cell dysfunction [97].

Our validation of *CLIC4* as a genuine miR-142-3p target provides one putative mechanism for CLIC4 regulation within tumors. We were encouraged by our identification of a reciprocal relationship between *CLIC4* and miR-142-3p expression, particularly in stage III/IV cervical SCC and HNSCC samples from TCGA, which is further supported by reports of stage-specific miR-142-3p upregulation during human bronchial squamous carcinogenesis [98]. Thus, miR-142-3p levels are highest in advanced squamous tumors where we previously noted the lowest levels of CLIC4 in tumor epithelium [23]. Several other studies report an association between high miR-142-3p levels in HNSCC and clinical features such as nodal invasion, poor prognosis, and reduced progression-free survival [99–102]. However, when considering that HNSCCs are among the most highly immune-infiltrated cancer types [103] and the known abundance of miR-142-3p within immune cells [50], we sought to determine the source of elevated miR-142-3p detected in bulk HNSCC tumors by leveraging the spatial resolution afforded by ISH. This revealed that miR-142-3p-expressing cells were localized predominantly to the stromal compartment. Immunostaining of serial tumor sections confirmed that CLIC4 expression was lower in tumor than stroma and that immune cell infiltrates positive for CD3 (T cells) and CD68 (monocytes) were abundant in the stroma. In other circumstances of miR-142-3p upregulation in bulk tissue, such as psoriasis [104] and intestinal allograft rejection [105], ISH has also shown that the source of miR-142-3p is infiltrating immune cells positive for markers of either T cells or monocytes/macrophages. Reports of dynamic evolution of tumor-infiltrating immune populations during HNSCC progression may also account for the stronger inverse relationship between *CLIC4* and miR-142-3p in advanced tumors if miR-142-3p is more strongly expressed or secreted by a distinct immune cell type [106]. Thus, the miR-142-3p upregulation described in certain cancers may be due to the presence of immune cells in tissue homogenates, which can only be distinguished by single-cell sequencing or *in situ* analysis. This, as well as the specific immune cell types expressing miR-142-3p, should be considered in future analyses, and those ascribing biomarker function to miR-142-3p should determine if it is a tumor cell-autonomous biomarker, a general marker of inflammation, or a marker of specific tumor-associated immune cell states, the latter of which could have implications for sensitivity to immunotherapy [107].

The contribution of stromal *CLIC4* to overall expression levels in bulk analysis was also made clear by our assessment of publicly available HNSCC scRNA-seq data [57]. Though we previously described reciprocal changes in CLIC4 expression between tumor epithelium and stroma during disease progression [23], this has never been quantified at the resolution made possible by scRNA-seq. We found that *CLIC4* expression was higher in tumor-associated fibroblasts and endothelial cells than in malignant tumor epithelial cells. This is supported by known roles of CLIC4 in supporting myofibroblast differentiation [11] and in endothelial cell proliferation and angiogenesis [4, 5]. *CLIC4* was notably absent from tumor-infiltrating T cells of all subtypes, despite prior documented expression in T cells [108]. Therefore, T cells, which are known to express miR-142-3p, may represent a cellular context in which miR-142-3p represses *CLIC4* to promote intrinsic phenotypic changes. CLIC4 is also highly expressed in other immune cells and is particularly abundant in activated macrophages [20]. At this time, it is not clear if the biological role for the interaction between *CLIC4* and miR-142-3p is to fine-tune gene expression within immune cells or if miR-142-3p is transferred from immune cells to tumor cells to downregulate *CLIC4* in a paracrine manner, but this will be the basis of future investigation.

Our finding that CLIC4 is expressed in several HNSCC cell lines *in vitro* but is downregulated upon shifting from culture to growth as *in vivo* xenografts suggests that *CLIC4* expression is altered by the transition to anchorage-independent growth or exposure to factors from the host microenvironment. Xue et al. also reported CLIC4 expression in HN4, an additional HNSCC cell line, but suggested that CLIC4 is elevated in HNSCC and its knockdown sensitizes HN4 cells to apoptosis [109]. However, we contend that the study design and exclusive use of *in vitro* manipulations, in light of the dynamic regulation of CLIC4 *in vivo*, does not tell the entire story. The influence of extracellular factors to modulate CLIC4 expression has been demonstrated for stromal fibroblasts, which depend on tumor cell-derived TGF-β for CLIC4 induction [11]. Our proposal that paracrine miR-142-3p is responsible for *CLIC4* downregulation in tumor epithelium is with the caveat that miR-142-3p could have the same effect on *CLIC4* in fibroblasts. While exosomes have been documented to have different tropisms for localization and uptake based on patterns of integrin expression [110], we have yet to determine if miR-142-3p is indeed released from immune cell exosomes into tumor or stromal fibroblasts in this context, or if the quantity would be sufficient to downregulate *CLIC4* to the extent observed within tumors. The degree of miRNA-mediated repression is dependent on both the miRNA and target gene expression level, resulting in either on/off switch-like regulation or more discrete fine-tuning [111]. Thus, cells such as tumor-associated fibroblasts, in which TGF-β drives strong upregulation of CLIC4 may be less sensitive to miRNA regulation of *CLIC4* than a cell with intermediate expression [11]. Based on our findings, we conclude that miR-142-3p is unlikely the sole cause of attenuated *CLIC4* expression in HNSCC tumor epithelium, but one of many regulators. Our initial miRNA screen also identified miR-122-5p as a regulator of CLIC4, and miR-122-5p was recently reported to be elevated in the saliva of patients with HNSCC [112]. Thus, other miRNAs and microenvironmental factors, including cytokines known to induce CLIC4 such as TGF-β and TNF-α, likely work in concert to modulate CLIC4 expression [11, 33, 113].

This is the first study to definitively demonstrate that *CLIC4* is not regulated by somatic mutation, copy number alteration, or promoter methylation in SCC. Our identification of *CLIC4* as a bona fide target of miR-142-3p has profound implications for future studies of CLIC4 biology, particularly if the interaction is identified in an endogenous context rather than following forced overexpression. Finally, our application of *in situ* and single-cell analysis to demonstrate discrete patterns of expression for both *CLIC4* and miR-142-3p underscores the fact that bulk expression data should be used with caution. Particularly, the cell of origin should be clearly identified for any molecule proposed as a biomarker, which is becoming more realistic in the modern era of rapidly improving and increasingly accessible single-cell technology.

## MATERIALS AND METHODS

### Immunohistochemistry

Immunohistochemistry was performed on serial sections from tissue microarrays (HN803e, BCN963b, and ES804) from US Biomax and cervical cancer specimens were provided by the Naval Medical Center San Diego. Immunohistochemistry for CLIC4 (Cell Signaling Technology Cat# 12644; RRID: AB_2797976) and αSMA (Cell Signaling Technology Cat# 19245; RRID: AB_2734735) was performed as described in our protocol at dx. doi. org/10.17504/protocols.io.2figbke. Immunohistochemistry for CD3, CD68, CD45, F4/80, and Ly6G was performed by the Pathology/Histotechnology Laboratory at the Frederick National Laboratory for Cancer Research. Brightfield images were obtained with an Aperio AT2 digital slide scanner followed by analysis with Aperio ImageScope software (Leica Biosystems).

### Bioinformatic analysis

The Cancer Genome Atlas (TCGA, RRID: SCR_003193) was queried using cBioPortal [39, 40] (RRID: SCR_014555) and EDGE in TCGA [41]. To assess *CLIC4* alteration frequency, all TCGA Pan Cancer Atlas studies were selected and filtered to include only those with both mutations and copy number alteration data (*n* = 9870). For EDGE analysis we accessed the EDGE R/Shiny application at http://ls-shiny-prod.uwm.edu/edge_in_tcga/ and queried the “gene-wise pan-cancer” tool for *CLIC4* (*n* = 3228). The UCSC Genome Browser (RRID: SCR_005780) was utilized to align ENCODE Regulation Transcription Factor ChIP tracks (RRID: SCR_006793), CpG islands, and other features at the *CLIC4* locus [42]. TargetScan (RRID: SCR_010845), DIANA-microT-CDS (RRID: SCR_016510), and miRmap (RRID: SCR_016508) were used to identify putative *CLIC4*-targeting miRNAs [45–47]. miRTarBase was used to assess fold change in expression for *CLIC4* and miR-142-3p in HNSCC vs. matched normal samples [52]. For expression correlation analysis, data from HNSCC and cervical SCC in TCGA were obtained and imported into the R statistical program [114] (RRID: SCR_001905) using the RTCGA package [115]: specifically, normalized miRNA-seq read counts, normalized RNA-seq read counts, and collated clinical data. Cancer staging was primarily determined with pathologic stage, as provided by TCGA, and supplemented with clinical stage as needed. Spearman’s correlation was used to evaluate the relationship between miR-142-3p and *CLIC4*. For *CLIC4* expression distribution analysis in HNSCC single cells, public scRNA-seq data from 18 patients [57] were downloaded from GEO (RRID: SCR_005012) accession number GSE103322. The t-distributed Stochastic Neighbor Embedding (t-SNE) method was applied for clustering of a subset of 2158 non-malignant cells processed with the Super Script II enzyme. The R Seurat package [116] was used to generate the t-SNE mapping with default parameters and principal component analysis (PCA) reduction with eight principal components. A Wilcoxon rank-sum test was performed to estimate differential upregulation or downregulation of *CLIC4* in a cluster vs. the other populations of cells or between malignant and non-malignant cell types (R Seurat *FindAllMarkers* function).

### Cell culture

293T cells were cultured in Dulbecco’s modified Eagle’s medium (DMEM) containing 10% fetal bovine serum (FBS; Gibco, Thermo Fisher Scientific) and penicillin/streptomycin. Human neonatal epidermal keratinocytes (HK; Lonza Cat# 00192906) were cultured in EpiLife medium (Thermo Fisher Scientific). SCC4 (ATCC Cat# CRL-1624; RRID: CVCL_1684), SCC9 (ATCC Cat# CRL-1629; RRID: CVCL_1685), SCC15 (ATCC Cat# CRL-1623; RRID: CVCL_1681), and SCC25 (ATCC Cat# CRL-1628; RRID: CVCL_1682) cells were obtained from the American Type Culture Collection (ATCC) and were cultured in DMEM/F12 (Mediatech, Corning Life Sciences) containing 10% FBS, penicillin/streptomycin, and 400 ng/mL hydrocortisone (Sigma-Aldrich). Cell morphology and confluence assessments were performed using an IncuCyte FLR live cell imaging system (Essen BioScience).

### Bisulfite sequencing

Genomic DNA was isolated from HK, SCC4, SCC9, SCC15, and SCC25 cells by incubating cell pellets overnight at 55°C in lysis buffer (100 mM NaCl, 10 mM Tris-Cl pH 8.0, 25 mM EDTA pH 8.0, 0.5% SDS, and 0.1 μg/μL proteinase K) followed by phenol/chloroform extraction and ethanol precipitation. Bisulfite conversion and purification of converted DNA was performed using a Bisulfite Conversion Kit (Active Motif) according to the manufacturer’s instructions. The test region was amplified by PCR using AmpliTaq Gold 360 (Applied Biosystems) with primers shown in Supplementary Table 4. PCR products were cloned using TOPO-TA cloning according to the manufacturer’s instructions (Thermo Fisher Scientific). Individual clones were isolated and sequenced by the CCR Genomics Core (National Cancer Institute, Bethesda, MD, USA) using an M13R primer (Thermo Fisher Scientific). Sequencing reads were trimmed in Sequencher (Gene Codes) and bisulfite conversion rate and methylated CpGs were determined using BiQ Analyzer (RRID: SCR_008423).

### Luciferase reporter assay

miTarget *CLIC4* 3′UTR target reporter and pEZX-MT05 control plasmids were obtained from Genecopoeia. The plasmids contain the *CLIC4* 3′UTR downstream of *Gaussia* luciferase (GLuc) or GLuc alone and secreted alkaline phosphatase (SEAP) for normalization. 293T cells were seeded in 48-well plates at 60,000 cells/cm^2^. The next day, cells were transfected using Lipofectamine 3000 (Thermo Fisher Scientific) according to the manufacturer’s protocol with 62.5 ng of reporter plasmid and miRNA mimics at a final concentration of 10–20 nM (Dharmacon) or target protector (QIAGEN) at 0.1–1 μM per well. Transfections were performed in antibiotic-free DMEM containing 10% FBS, and the medium was changed 24 hours after transfection. Supernatants were harvested at 48–72 hours after transfection. GLuc and SEAP activity were quantified using the Secrete-Pair Dual Luminescence Assay Kit (Genecopoeia) according to the manufacturer’s protocol. Luminescence was detected using an Infinite M200 plate reader (Tecan).

### Western blotting

Cell lysates were prepared using radioimmunoprecipitation assay (RIPA) buffer (Cell Signaling Technology) containing protease inhibitors (Thermo Fisher Scientific). Protein concentrations were determined using the Pierce BCA Protein Assay Kit (Thermo Fisher Scientific). Equal amounts of protein were resolved on 4–20% Criterion TGX gels (Bio-Rad) and transferred to nitrocellulose membranes using the Trans-Blot Turbo Transfer System (Bio-Rad). Membranes were blocked in 5% milk dissolved in tris-buffered saline with 0.1% Tween 20 (TBST) for 1 hour at room temperature. Membranes were then incubated with primary antibody diluted in 3% bovine serum albumin (BSA) in TBST at 4°C overnight, followed by washing in TBST, incubation with HRP-conjugated goat anti-rabbit secondary antibody at 1:10,000 (Cell Signaling Technology Cat# 7074, RRID: AB_2099233) in 3% BSA/TBST for 1 hour at room temperature, and a final set of washes in TBST. Proteins were detected using the SuperSignal West Pico Chemiluminescent Substrate (Thermo Fisher Scientific) and visualized using a ChemiDoc Touch Imager (Bio-Rad). Primary antibodies and dilutions included CLIC4 at 1:3000 (Cell Signaling Technology Cat# 12644, RRID: AB_2797976), HSP90 at 1:3000 (Cell Signaling Technology Cat# 4877, RRID: AB_2233307), TGFBR1 at 1:500 (Abcam Cat# ab31013, RRID: AB_778352), and p21 at 1:1000 (Cell Signaling Technology Cat# 2947, RRID: AB_823586).

### Site-directed mutagenesis

Site-directed mutagenesis of the *CLIC4* 3′UTR reporter plasmid was performed using the QuikChange Lightning Site-Directed Mutagenesis Kit (Agilent) according to the manufacturer’s protocol. Mutagenesis primer sequences are shown in Supplementary Table 4.

### Mimic and inhibitor transfection

SCC4 cells were seeded at 10,000/cm^2^ and SCC9, SCC15, and SCC25 cells were seeded at 20,000/cm^2^ in 6-well plates. The next day, control mimic (Dharmacon), miR-142-3p mimic (Dharmacon), or *CLIC4* siRNA (QIAGEN) were transfected at 20 nM using Lipofectamine 3000 (Thermo Fisher Scientific) according to the manufacturer’s instructions. For inhibitor experiments, 25–100 nM control inhibitor (Dharmacon) or miR-142-3p inhibitor (Dharmacon) was transfected into SCC4 cells with Attractene (QIAGEN) according to the manufacturer’s instructions. Dishes were rinsed with PBS and snap frozen for later protein extraction at 48–72 hours after transfection.

### *In situ* hybridization

Serial sections of a human HNSCC tumor tissue microarray (HN802e) were obtained from US Biomax. miRNA *in situ* hybridization was performed by Multiplex DX (Gaithersburg, MD, USA) with a double-DIG-labeled locked-nucleic acid miR-142-3p probe (QIAGEN) or control scrambled probe as previously described [117]. Fluorescent images were obtained using a Nikon ECLIPSE Ti2 microscope and an identical exposure time and intensity settings were used for all images.

### Quantitative reverse transcription PCR

RNA was extracted using the miRNeasy Mini Kit and the QIAcube platform (QIAGEN). mRNA was reverse transcribed using the iScript cDNA Synthesis Kit (Bio-Rad). For miRNA, gene-specific reverse transcription was performed as described by Kramer 2011 [118] using NxGen M-MuLV Reverse Transcriptase, NxGen RNAse Inhibitor, and PCR Grade dNTPs (Lucigen). PCR was performed with iQ SYBR Green Supermix (Bio-Rad). Primer sequences are shown in Supplementary Table 4. Thermal cycling and detection were performed using the CFX Connect Real-Time PCR Detection System (Bio-Rad) and data were processed using CFX Manager software (Bio-Rad).

### SCC xenografts

Mouse studies were performed under a protocol approved by the National Cancer Institute and the National Institutes of Health Animal Care and Use Committee. SCC4 cells (1 × 10^6^) or SCC25 cells (2 × 10^6^) were injected intradermally into the back of 6-12-week-old female athymic nude mice. Tumors were harvested when they reached at least 200 mm^3^. Portions of each tumor were flash frozen for subsequent biochemical analysis or fixed in 10% neutral buffered formalin (Fisher Scientific) followed by paraffin embedding and sectioning (Histoserv, Germantown, MD, USA).

### Statistical analysis

All cell line and xenograft data were analyzed using GraphPad Prism. For data with more than two groups, significance was determined using an analysis of variance (ANOVA) test with Dunnett’s correction for multiple comparisons. For data with two groups, significance was determined using an unpaired Student’s *t*-test. ^*^ *p* ≤ 0.05, ^**^ *p* ≤ 0.01, ^***^ *p* ≤ 0.001, ^****^ *p* ≤ 0.0001.

## SUPPLEMENTARY MATERIALS









## Abbreviations

ACD: AML/RUNX, CLIC, DSCR/RCAN
ADC: adenocarcinoma
AP-1: activator protein 1
αSMA: alpha smooth muscle actin
ATF: activating transcription factor
bp: base pair
BMDM: bone marrow-derived macrophage
CAF: cancer-associated fibroblast
CCLE: cancer cell line encyclopedia
CCR: Center for Cancer Research
CEBPB: CCAAT enhancer binding protein beta
ChIP: chromatin immunoprecipitation
CK2: casein kinase 2
CLIC: chloride intracellular channel
CTCF: CCCTC-binding factor
CREB: cyclic AMP response element-binding protein
DHS: DNase hypersensitivity site
EDGE: exploring drivers of gene expression
ENCODE: encyclopedia of DNA elements
EV: extracellular vesicle
FC: fold-change
GEO: gene expression omnibus
GLuc: *Gaussia* luciferase
HNSCC: head and neck squamous cell carcinoma
IHC: immunohistochemistry
ISH: *in situ* hybridization
LPS: lipopolysaccharide
MAX: MYC-associated factor X
μM: micromolar
miRNA/miR: microRNA
NCI: National Cancer Institute
NF-κB: nuclear factor kappa-light-chain-enhancer of activated B cells
nM: nanomolar
NRF1: nuclear respiratory factor 1
OCT4: octamer-binding transcription factor 4
PAX5: paired box 5
PKC: protein kinase C
qPCR: quantitative polymerase chain reaction
RCAN: regulator of calcineurin
scRNA-seq: single-cell RNA sequencing
SEAP: secreted alkaline phosphatase
SCC: squamous cell carcinoma
siRNA: small interfering RNA
SOX2: SRY-box transcription factor 2
TCF3: transcription factor 3
TCGA: The Cancer Genome Atlas
TGF-β: transforming growth factor beta
TLR: toll-like receptor
TNF-α: tumor necrosis factor alpha
TP: target protector
TP53: tumor protein 53
t-SNE: t-distributed Stochastic Neighbor Embedding
UCSC: University of California, Santa Cruz
UTR: untranslated region
