# Supplementary material for: Head and neck squamous cancer progression is marked by CLIC4 attenuation in tumor epithelium and reciprocal stromal upregulation of miR-142-3p, a novel post-transcriptional regulator of CLIC4
Source: Oncotarget. 2019 Dec 31;10(68):7251–75. doi: 10.18632/oncotarget.27387 (PMC6944452; doi:10.18632/oncotarget.27387)
Supplement: Supplementary file 1 [file oncotarget-10-7251-s001.pdf]

Head and neck squamous cancer progression is marked by CLIC4 attenuation in tumor epithelium and reciprocal stromal upregulation of miR-142-3p, a novel post-transcriptional regulator of *CLIC4*

SUPPLEMENTARY MATERIALS

Supplementary Table 1: *CLIC4* alterations detected in TCGA PanCancer Atlas. See Supplementary Table 1

Supplementary Table 2: Putative miRNAs targeting *CLIC4*. See Supplementary Table 2

Supplementary Table 3: scRNA-seq top 50 markers per cluster and *CLIC4* fold-changes per cluster and in malignant vs. non-malignant cells. See Supplementary Table 3

Supplementary Table 4: Primer sequences used in this study

Name	Forward Sequence	Reverse Sequence	Final concentration in reaction	Purpose
AMP2	GGTYGAGGTA TGAGGTTATTT GGTATAGTAATAG	AACTAACCACRAC TTCAACTCCTCAACACC	480 nM	Amplify bisulfite sequencing amplicon
Mut1	CTATCTACAAAGCA ATATAGCAAAATCTC ATGAGACTTTTGTAG AAAGCTCTCTTCTCTGG	CCAGAGAAGAGAG CTTTCTACAAAAGT CTCATGAGATTTTGC TATATTGCTTTGTAGATAG	125 ng	Mutagenize site 1 in CLIC4 3′UTR reporter plasmid
Mut2	TATAGCTAAGTCAACTA AAAATCAGTTATCATGAG TTGCTCAACAGATGTACA AATAGCAAGC	GCTTGCTATTTGTAC ATCTGTTGAGCAAC TCATGATAACTGAT TTTTAGTTGACTTA GCTATA	125 ng	Mutagenize site 2 in CLIC4 3′UTR reporter plasmid
Mut3	TA CATTTCTATTTCCTCTA CATTTTTACTTTCATGATA TTTTCTAAATGGGTTTAA GGGCACAGAAATAAATG	CATTTATTTCTGTGC CCTTAAACCCATTTA GAAAATATCATGAAA GTAAAAATGTAGAGG AAATAGAAATGTA	125 ng	Mutagenize site 3 in CLIC4 3′UTR reporter plasmid
RNU6-RT	-	CGCTTCACGAATTTG CGTGTCAT	100 nM	Gene-specific reverse transcription
miR-142-3pSL	-	GTCGTATCCAGTGCAG GGTCCGAGGTATTCGC ACTGGATACGACTCCATA	500 nM	Gene-specific reverse transcription
RNU6	GCTTCGGCAGCACAT ATACTAAAAT	CGCTTCACGAATTTGCGTG TCAT	250 nM	Quantitative PCR
HsRPL37A	ATTGAAATCAGCCA GCACGC	AGGAACCACAGTGCCAG ATCC	250 nM	Quantitative PCR
HsCLIC4	CAGACCTGCAGAA CTTGGCT	GCTTTAAGTACTTGGGAG GGCA	250 nM	Quantitative PCR
miR-142-3p	CGCGCCTGTAGTGT TTCCTACTTT	CCAGTGCAGGGTCCG AGGTA	250 nM	Quantitative PCR
